# Systematic Review of Studies Examining Transtibial Prosthetic Socket Pressures with Changes in Device Alignment

**DOI:** 10.1007/s40846-017-0217-5

**Published:** 2017-01-21

**Authors:** Philip Davenport, Siamak Noroozi, Philip Sewell, Saeed Zahedi

**Affiliations:** 1 Department of Design and Engineering, Bournemouth University, Poole, UK; 2 Bournemouth University, Poole, UK; 3 Chas A Blatchford and Sons Ltd., Basingstoke, UK

**Keywords:** Below-knee, Prostheses, Misalignment, Normal stress, Pressure distribution

## Abstract

Suitable lower-limb prosthetic sockets must provide an adequate distribution of the pressures created from standing and ambulation. A systematic search for articles reporting socket pressure changes in response to device alignment perturbation was carried out, identifying 11 studies. These were then evaluated using the American Academy of Orthotists and Prosthetists guidelines for a state-of-the-science review. Each study used a design where participants acted as their own controls. Results were available for 52 individuals and five forms of alignment perturbation. Four studies were rated as having moderate internal and external validity, the remainder were considered to have low validity. Significant limitations in study design, reporting quality and in representation of results and the suitability of calculations of statistical significance were evident across articles. Despite the high inhomogeneity of study designs, moderate evidence supports repeatable changes in pressure distribution for specific induced changes in component alignment. However, there also appears to be a significant individual component to alignment responses. Future studies should aim to include greater detail in the presentation of results to better support later meta-analyses.

## Introduction

Suitable pressure distribution within the lower-limb prosthetic socket is important for the comfort and function of the amputee [1–3], and an adequately fitting socket is required for extensive use of a functional prosthesis [4]. Inappropriate sockets have been implicated in cases of dermatological issues [5, 6] and pressure injury [7, 8]. Furthermore, socket comfort and socket fit are widely cited by users as the most important factor in their satisfaction with a prosthetic lower limb [9].

Different design philosophies exist in the production of transtibial prosthetic sockets. From the early 1960s, patellar tendon bearing (PTB) sockets became commonplace—in these designs, the socket is crafted to selectively load areas that are load-tolerant while at the same time offloading regions where applied pressure can be painful. Later, total surface bearing (TSB) designs were introduced, where the load is more evenly distributed around the entire residual limb. This was developed further into hydrostatic designs, where an equally applied hydraulic or pneumatic pressure is used to the form a socket shape where the residual limb tissues are forced to ‘flow’ into a configuration with equal pressure distribution. Clearly the aims of establishing a pressure distribution vary in each case, however in all cases a well-fitting socket is considered crucial for the successful use of a prosthetic limb [10].

A related aspect of device set-up is in the alignment of the device with the residual limb. Prosthetic limbs are adjustable in both rotation and translation in each plane, and poor alignment has been shown to affect multiple aspects of the gait of transtibial amputees [11–14]. Alignment has long been theorised to produce systematic changes in the pressure distribution at the socket interface [15]. Although amputees are able to tolerate a range of acceptable alignments [16], and different prosthetists are able to generate acceptable alignment geometries that may not necessarily match [17], creating suitable sockets and acceptable prosthesis alignment can be challenging even for experienced prosthetists [18]. The range of a satisfactory device alignment is thought to reduce when challenges to walking conditions are introduced [19].

Transtibial amputees form the largest group using functional prostheses that require alignment [20], and with a well-set up prosthesis can often be restored to near-normal walking efficiency [21]. The effects of alignment perturbation in transtibial amputees were most recently reviewed by Neumann [22] for a range of biomechanical characteristics including changes in pressure distribution. They concluded that alignment modification could induce meaningful changes in joint kinematics, kinetics and socket pressures, as well as the indication that ranges of alignments prove acceptable to users but that this range shows inter-subject variability.

Despite the importance of the effects of device alignment on pressure distribution and hence the functional ability of amputees, detailed evaluation of the measurement techniques in use matched to studies’ internal and external threats to validity has not been carried out. As the process of supplying sockets and identifying suitable alignment remains a clinically relevant issue, and given the range of measurement methods in use and that new techniques have been introduced in recent years (e.g., fibre-Bragg grating sensors [23], inverse problem neural networks [24] and 3D printed capacitance sensors [25]), an updated evaluation of issues in pressure measurement studies with changes in prosthetic alignment will be of use to researchers.

**Fig. 1:**
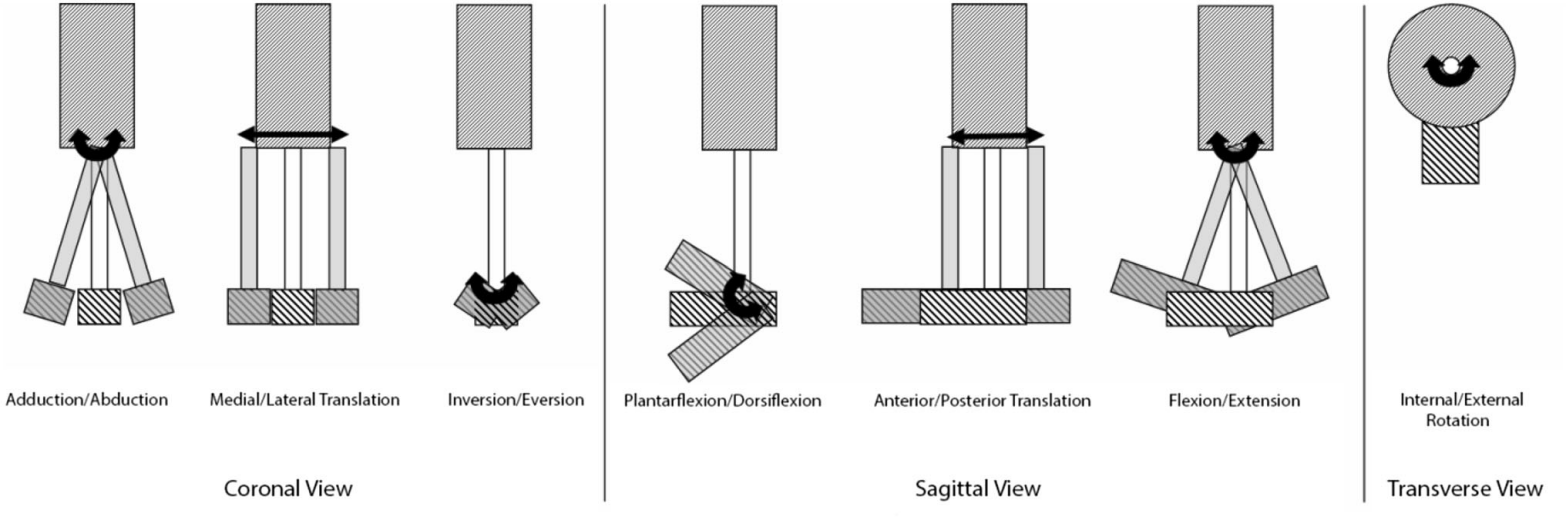
Potential alignment modifications to transtibial prostheses. The eighth possible adjustment—changes to pylon height—is not shown (and was not investigated by any included articles)

In Neumann’s review of alignment effects in transtibial amputees, eight potential perturbation methods were defined. As these designations form a complete and concise description of alignment changes, they have been used to classify interventions in the included studies and illustrated here for clarity. These are socket flexion/extension (sagittal plane rotation of the socket), abduction/adduction (coronal plane rotation of the socket), anterior/posterior translation (sagittal plane translation of the foot relative to the socket), medial/lateral translation (coronal plane translation of the foot relative to the socket), plantarflexion/dorsiflexion (sagittal plane rotation at the ankle), inversion/eversion (coronal plane rotation at the ankle), internal/external rotation (transverse plane rotation of the ankle) and pylon length (Fig. 1). However, that investigation identified numerous gaps in the assessment of these alignment adjustments.

The objectives of this study were to perform an updated systematic search for studies that measured transtibial socket pressure changes with any changes in prosthesis alignment during walking, to report the measurement methods in use and to evaluate these studies for common threats to validity in greater detail than previously. From the included studies, evidence statements will be obtained and graded according to strength of support, and the implications for best practice in socket pressure research discussed. This will help researchers understand the limitations of previous work, and aid clinicians in evaluating the results from such measurements.

The effect of alignment modification on changes in shear force (i.e., forces in the plane parallel to the socket/stump interface) has not been considered in this study. Shear forces are believed to be an important component in evaluating the risk of pressure injury and, like normal stress, are associated with the quality of socket fit [26]. However measuring such forces is only possible using a limited range of measurement transducers [27], and hence has been studied even less frequently than normal pressures [22]. Although transducers are being developed which may enable the more convenient collection of shear pressures [28, 29], the current generation of sensors do not in general permit this. For these reasons, an evaluation of the conclusions of shear measurement studies was not completed.

## Methods

The review was carried out using the American Academy of Orthotists and Prosthetists (AAOP) guidance for creating a state-of-the-science review [30]. This was chosen as these guidelines were developed with consideration for the expected qualities of prosthetics studies. In addition, it is better suited for analysis of studies that are not randomised-controlled trials: these are uncommon in the amputee literature where the majority of interventions are reported as case series or case-controlled studies. For these reasons this was selected as a tool ahead of similar methods such as the preferred reporting items for systematic reviews and meta-analyses guidelines [31].

### Eligibility Criteria

Eligibility criteria for studies are described in Table 1 [for participant–intervention–comparison–outcome-study type (PICOS) characteristics] and in Table 2 (for details of acceptable report characteristics). Any reports whose full text was not available through the author’s institution library system would also be rejected, but no studies identified proved unavailable.

**Table 1:** PICOS framework for inclusion of eligible studies

Section	Criteria
Participants	• Unilateral transtibial amputees • Any prescription of functional prosthesis (excluding osseointegration) • Any cause for amputation (e.g., trauma, vascular conditions or other)
Intervention	• Any (single or combination) of translation or rotation of prosthetic components that altered the geometric position of the artificial foot relative to the residual limb
Comparison	• Between altered alignment states and ‘normal’ or neutral’ conditions
Outcome	• Quantitative measurement of socket-residuum pressure (normal stress) • Any mechanism for achieving this measurement
Study type	• Any primary research, including case series or case studies

**Table 2:** Eligible report characteristics

Section	Criteria
Language	• Studies published in English
Publication type	• Peer reviewed journal articles of primary research (i.e., excluding literature reviews, letters to the editor, commentaries, etc.)
Publication date	• Database inception: April 2016

### Information Sources

Four databases were searched, with full details and search strings used included in Table 3. The databases selected indexed each of the journals reported by the AAOP guidelines as being the most common sources of relevant studies.

**Table 3:** Database search strategy employed in this study

Database	Search string
Web Of Science	(pressure* OR stress*) AND (angle OR *align* OR angular) AND (prosthe*) AND (transtibial OR trans-tibial OR below-knee OR “below knee”)
CINAHL	TX (pressure* OR stress) AND TX (angle OR *align* OR angular) AND TX prosthe* AND TX (transtibial OR trans-tibial OR below-knee OR “below knee”), Academic Journals Only
ScienceDirect	(trans$tibial OR “trans tibial” OR “below knee” OR below$knee) AND (pressure* OR stress*) AND (angle OR *align* OR angular) AND (prosthe*)
RECAL Legacy^a^	pressure AND alignment AND below knee

* A truncation character, $ a wildcard character. TX indicates terms were extended to the full text of records

^a^Indexing to the RECAL database concluded in 2007, and so results were available from inception to 2007 only

### Study Selection

After duplicates were removed, the titles of each paper were reviewed by the lead author. Titles were rejected if they clearly did not refer to lower limb prosthetics or were not in English. The abstracts of the remaining papers were assessed for relevance. Finally, the full text of the remaining articles were obtained and appraised. Five articles were removed at this stage: two papers dealt with standing pressure changes only [32, 33], one reported experimental results in reference to an FEA model only [34], and one did not include transtibial participants [35]. One study which had been included in previous reviews was rejected from this study as only an abstract had been published and which lacked sufficient information for further analysis [36].

**Fig. 2:**
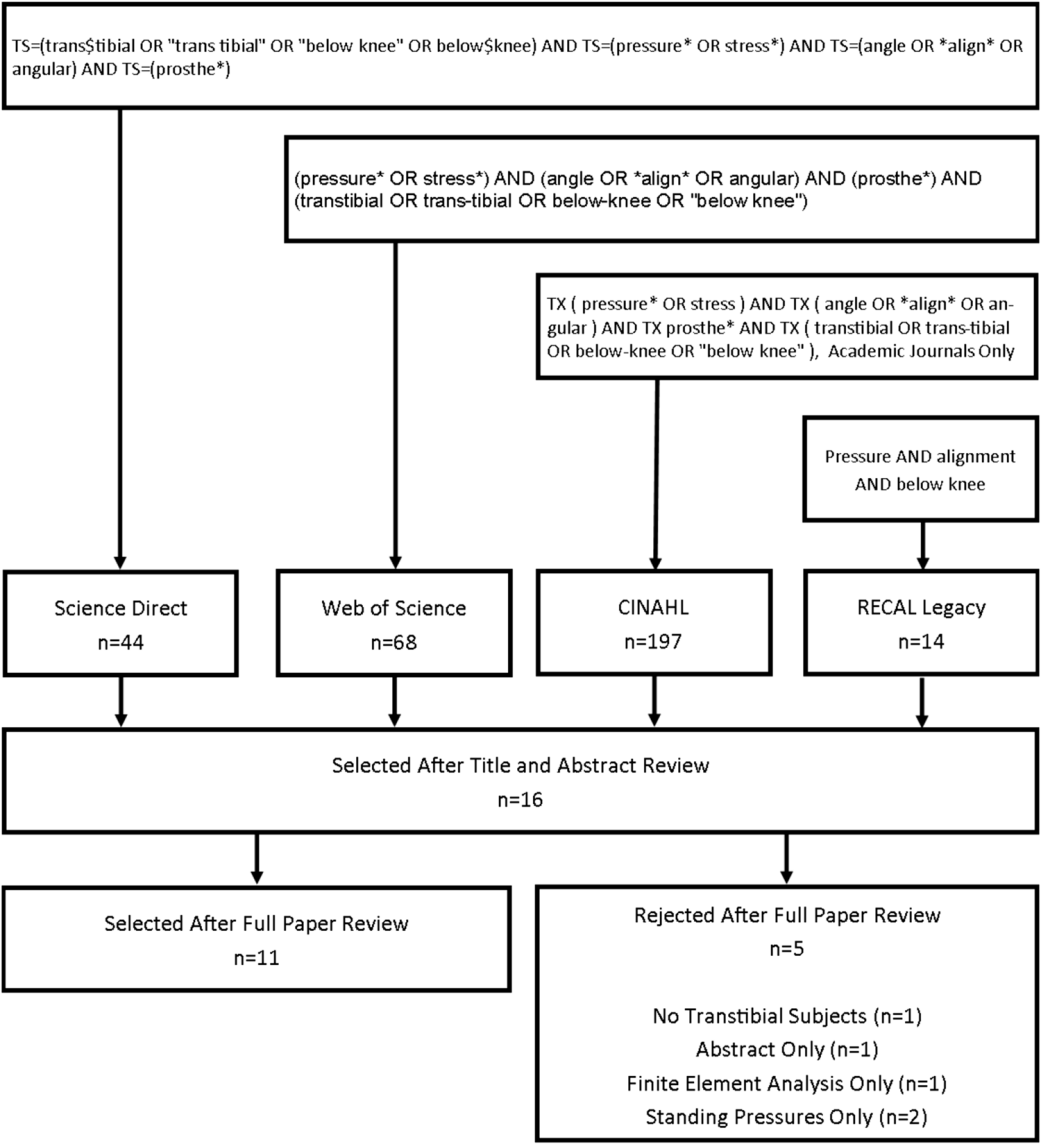
Flowchart of the literature search and study selection process

Finally, the reference list of each included paper was examined for any remaining relevant articles which had not been indexed in the search process—this did not identify any additional studies. The process is summarised in Fig. 2.

### Data Collection Process

The 11 selected papers were assessed for methodological quality using a modified AAOP quality assessment form similar to that used by Neumann [22]. This identified the sources of risks to internal and external validity in each study. This was modified to better support the aims of the current study and the focus on pressure studies alone. The full description of each question is included in Appendix.

### Data Items

Details of the measurement instrumentation, the alignment intervention and the positions of pressure measurement were examined. Statistical methods, where these were reported, were also appraised.

Confidence in the conclusions of each included study was evaluated by the lead author. Using a simple numerical scale to determine this is deprecated by the AAOP guidelines in favour of an approach that weights more heavily the critical aspects of study design and analysis. Internal and external threats to validity were considered separately. Confidence was expressed as low, moderate or high as described in Table 4.

**Table 4:** Definition of confidence levels in conclusions

Rating	Description
High	High confidence can be placed in the findings of this investigation. The article is methodologically strong, or has methodological issues that are unlikely to impact the confidence with which the outcome statement can be made. Tests of statistical significance have been undertaken
Moderate	Moderate confidence can be placed in the findings from this investigation. There are some methodological issues that detract from our confidence in the findings
Low	Low confidence can be placed in the findings from this investigation. There are significant methodological issues which compromise the confidence with which outcome statements can be made

For internal validity, high quality studies required calculations of statistical significance, blinding with adequate randomisation and a complete description of the alignment intervention and measurement method. Moderate reliability is indicated by the presence of adequate descriptive statistics (i.e., some average and variance) of the outcome method, but also that some aspect of the repeatability of the study is flawed such that high confidence cannot be assigned to the conclusions. Low confidence is reported if descriptions of the participants or intervention were lacking or if interventions are not randomised or blinded in any fashion.

External validity was assessed in a similar manner. High validity scores required suitable statistical calculations, complete descriptions of participants and discussion that placed the results in the context of other studies and biomechanical expectations. In moderate-rated studies, the above attributes were lacking in some way: either no statistical significance was calculated or discussion was inadequate for example. Low confidence was assigned if data was missing from the report or if descriptions of the participants were substantially incomplete.

Based on the internal and external validity ratings of each study and the strength of the conclusions drawn, a list of evidence statements arranged by the degree of confidence in veracity was produced. The conclusions were rated using the confidence levels described in Table 4.

## Results

A summary of the 11 selected studies is included in Table 5, including the lead author and year of publication, the number and gender balance of participants, details of the measurement method and the alignment interventions that were studied.

**Table 5:** Description of all studies meeting the inclusion criteria

Lead authors	Years	Participants	Gender	Measurement sites	Transducer types	Collection frequency	Intervention
Pearson et al. [37]	1973	10^a^	10M	Patellar tendon, distal anterior tibia, lateral/medial tibia	Diaphragm SG	NR	A/P −10/−5/0/5/10 mm M/L −10/−5/0/5/10 mm F/E −10/−5/0/5/10° Ab/Ad −10/−5/0/5/10°
Winarski and Pearson [38]	1987	2	NR	Patellar tendon, gastrocnemius	Diaphragm SG	200 Hz	F/E −10/−6/−3/0/3/6/10°
Sanders et al. [39]	1993	3	3M	Antero-medial proximal, antero-lateral–distal, antero-medial–distal–lateral, postero-proximal, postero-distal	Piston SG	125 Hz	Ankle DF/PF 6/0/−9°
Sanders et al. [40]	1998	2	2M	Antero-lateral–distal, antero-lateral–medial, antero-lateral-mid, antero-medial-mid, antero-lateral–proximal, antero-medial proximal, lateral–distal, lateral–proximal–distal, lateral-mid, lateral–proximal, posterior distal, posterior-mid, popliteal fossa	Piston SG	175 Hz	Subject specific A/P, M/L translation Ab/Ad rotation Ankle DF/PF
Zhang et al. [41]	1998	1^b^	NR	Lateral condyle, medial condyle, patellar tendon, lateral tibia, medial tibia, antero-distal, popliteal depression, medial gastrocnemius, lateral gastrocnemius	Piston SG	200 Hz	F/E −8/0/8°
Sanders and Daly [42]	1999	3	3M	As in Sanders et al. [39]	Piston SG	125 Hz	Subject specific Ankle DF/PF
Seelen et al. [43]	2003	17	11M, 6F	Array measurement on anterior, medial and lateral aspects	Point FSR	50 Hz	5 mm heel and forefoot wedging
Kang et al. [44]	2006	10	NR	Array measurement	Array FSR	NR	F/E 10/5/0°
Jia et al. [45]	2008	1	1M	Array measurement	Array FSR	50 Hz	F/E −6/0/6°
Neumann et al. [46]	2013	2^c^	1M, 1F	Array measurement, regions selected on patellar tendon, popliteal depression, distal tibia and gastrocnemius	Array FSR	200 Hz	A/P −5/0/5 mm
Courtney et al. [47]	2016	1	1M	Array measurement	Array FSR	NR	A/P −10/0/10 mm M/L −10/0/10 mm F/E −3/0°

*NR* not reported, *A*/*P* anterior–posterior, *M*/*L* medial–lateral, *F*/*E* flexion–extension, *Ab*/*Ad* abduction–adduction, *DF*/*PF* dorsiflexion–plantarflexion, *SG* strain gauge, *FSR* force sensitive resistor

^a^Of the 10 participants, results for coronal plane alignment changes were reported for a single subject only

^b^Although Zhang et al. recruited five participants, only one took part in alignment alteration measurements

^c^Neumann recruited four participants; however equipment failure meant that results for only two were available

**Fig. 3:**
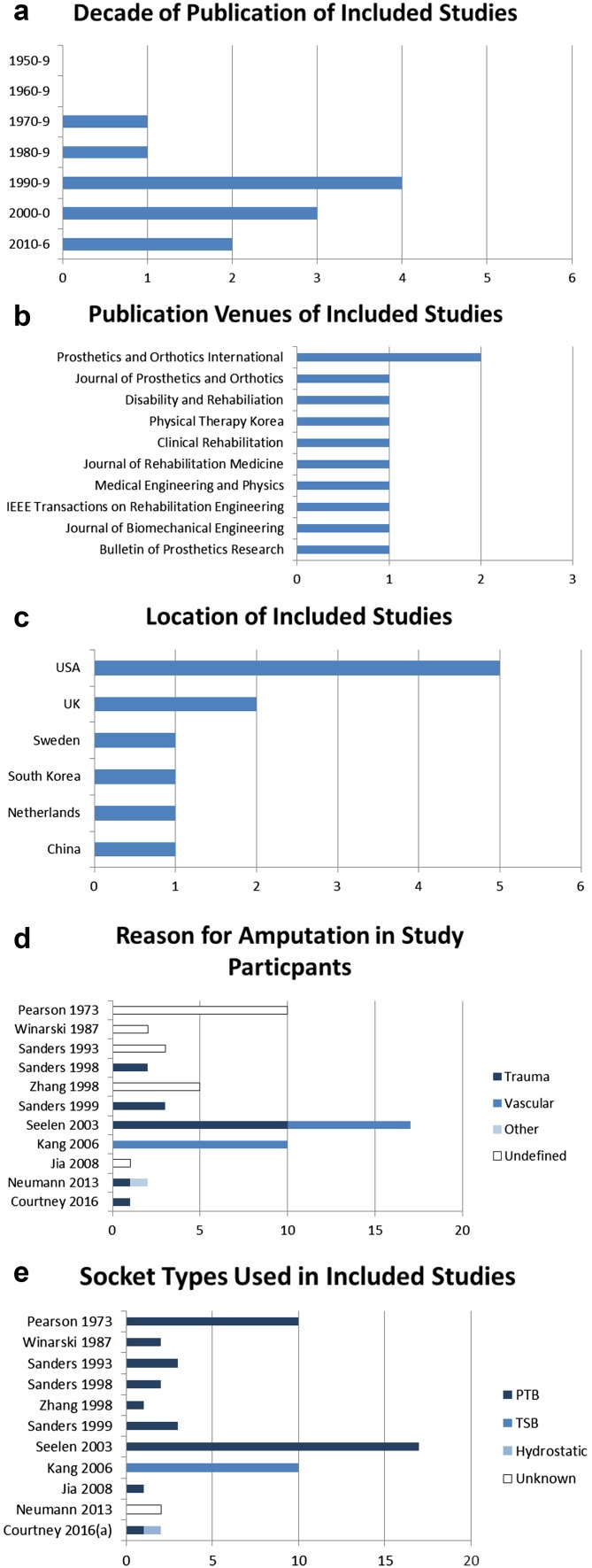
**a** Distribution of date of publication of selected studies. **b** Publication venue of included studies. **c** Nationality of study participants in included reports. **d** Reasons for amputation in study participants in included reports. Kang 2006 is reported as vascular although the article reports that cause was “vascular disease such as trauma or diabetes mellitus”. **e** The tested socket design of study participants in included reports. *PTB* patella tendon bearing, *TSB* total socket bearing. Sanders 1999 used participants who habitually used PTB sockets, but test prostheses were manufactured using computer aided design and manufacturing. Courtney 2016 is reported as one PTB and one hydrocast socket as both designs were tested on a single participant

Studies were identified from a wide range of journals, locations and times (Fig. 3a–c). The earliest was published in 1973 and the most recent in 2016. Ten different journals published studies: the only journal with more than one publication was *Prosthetics and Orthotics International*: many venues were not included on the AAOP list of common sources for prosthetic research. Publications included participants worldwide, but predominately from developed medical systems, and the majority from the USA and UK.

All studies were classified as controlled before and after studies (i.e., experimental studies where individuals acted as their own controls to the intervention being studied). Four studies were rated as providing moderate confidence in both their internal and external validity—although it was possible for internal and external validity ratings to differ, this was not the case in this study.

The particular concerns of study validity are presented in Tables 6 and 7.

**Table 6:** Internal validity scoring

Lead authors	Years	IV6			IV7			IV8				IV9		
		a	b	c	a	b	c	a	b	c	d	a	b	c
Pearson	1973	x			x		x	x	x			x	x	x
Winarski	1987	x			x			x				x	x	x
Sanders	1993	x										x		
Sanders	1998			x						x		x		
Zhang	1998				x			x				x	x	
Sanders	1999	x			x			x				x		
Seelen	2003	x					x					x		x
Kang	2006	x							x			x		x
Jia	2008			x					x			x		x
Neumann	2013			x								x	x	
Courtney	2016			x	x			x	x			x		x

Lead authors	Years	IV10		IV11		IV12	IV13							
		a	b	a	b	a	a	b	c	d	e	f	g	h
Pearson	1973	x								x	x			x
Winarski	1987	x					x		x	x				
Sanders	1993	x					x		x	x	x			
Sanders	1998		x				x		x	x				
Zhang	1998	x					x		x	x				
Sanders	1999	x					x		x	x	x	x		
Seelen	2003	x					x		x	x		x		
Kang	2006	x							x	x	x		x	x
Jia	2008		x				x		x	x	x	x		
Neumann	2013	x			x		x		x					
Courtney	2016	x					x		x	x		x	x	x

Lead authors	Years	IV14		IV15	IV16		IV17	IV18	IV19		Sum
		a	b	a	a	b	a	a	a	b	
Pearson	1973		x	x	x		x				16
Winarski	1987		x	x	x		x			x	15
Sanders	1993		x	x			x			x	11
Sanders	1998			x						x	9
Zhang	1998		x	x	x		x				12
Sanders	1999			x	x						12
Seelen	2003			x					x	x	12
Kang	2006			x		x					12
Jia	2008		x	x	x		x				14
Neumann	2013		x	x	x		x			x	12
Courtney	2016		x	x	x		x			x	18

x indicates the presence of a concern within a particular study. Each assessment criteria is described in Appendix

**Table 7:** External validity scoring

Lead authors	Years	EV1	EV2			EV3			EV4			EV5	
		a	a	b	c	a	b	c	a	b	c	a	b
Pearson	1973	x	x	x	x	x	x	x	x	x			
Winarski	1987	x	x	x	x	x	x	x	x	x		x	
Sanders	1993	x	x	x	x	x		x	x	x		x	
Sanders	1998		x		x					x	x	x	
Zhang	1998	x	x			x	x	x	x	x		x	
Sanders	1999		x	x	x		x		x	x		x	
Seelen	2003	x	x		x				x	x		x	
Kang	2006	x	x	x						x			
Jia	2008		x	x	x	x	x			x		x	
Neumann	2013		x	x		x	x	x	x	x		x	
Courtney	2016		x	x		x	x	x		x		x	

Lead authors	Years	EV6						EV7		EV8			Sum
		a	b	c	d	e	f	a	b	a	b	c	
Pearson	1973		x	x						x	x		13
Winarski	1987		x	x	x	x							14
Sanders	1993	x	x	x									12
Sanders	1998		x	x		x	x		x				10
Zhang	1998		x	x		x		x					12
Sanders	1999	x	x	x		x	x		x				13
Seelen	2003		x	x				x				x	10
Kang	2006		x	x		x			x				8
Jia	2008		x	x		x			x				11
Neumann	2013		x	x				x					11
Courtney	2016		x	x		x		x	x				12

x indicates the presence of a concern within a particular study. Each assessment criteria is described in Appendix

## Discussion of Study Quality

The articles identified exhibited a range of threats to their internal and external validity. In terms of internal threats, the range of scores was from 9 (Sanders et al. [39]) to 18 (Courtney et al. [47]) out of a possible 30. No papers were considered to have a high confidence in their internal validity, and only four were regarded as having moderate confidence (Table 8). Particular qualities are described here.

**Table 8:** Internal and external validity ratings of each included study

Lead authors	Years	Internal validity rating	External validity rating
Pearson	1973	Low	Low
Winarski	1987	Low	Low
Sanders	1993	Low	Low
Sanders	1998	Moderate	Moderate
Zhang	1998	Low	Low
Sanders	1999	Moderate	Moderate
Seelen	2003	Moderate	Moderate
Kang	2006	Moderate	Moderate
Jia	2008	Low	Low
Neumann	2013	Low	Low
Courtney	2016	Low	Low

The majority of studies included no blinding to intervention of any kind. Four studies blinded participants to alignment changes. Only one study (Zhang et al. [41]) reported double-blinding. It has been suggested that fully effective blinding of participants is unlikely in prosthesis configuration studies; however there is some evidence to suggest that transtibial amputees have only a limited ability to detect changes in device alignment [18], in particular changes that are small in magnitude. Despite this, even nominal blinding of participants was not attempted in most cases (although the order of alignment change was reported as randomised in most studies). Investigators were blinded in four studies: typically that the alignment changes are carried out in a random order, and physically altered by a separate member of the investigation team.

As far as could be determined, all studies recruited using samples of convenience. This meant that although inclusion criteria were generally well established and well reported, exclusion criteria were more poorly described. The sockets used in studies were often not well evaluated in terms of quality of fit.

Where the reason for amputation was reported, the number of participants was evenly split between amputation as a result of trauma and for dysvascular reasons (Fig. 3d). It should be noted that Kang 2006 described their participants as having amputation “as a result of vascular reasons, such as trauma or diabetes mellitus”, a clearly contradictory description. The number may be further biased to traumatic amputees. In a related note to the use of samples of convenience, the large number of cases where amputations reason was undefined (in particular in early studies) can be assumed to have been participants with traumatic amputation due to the typically younger age and greater walking ability predisposing them to research participation. The issue of representativeness common to many fields of prosthetic study [48], but these results indicate that vascular amputees (who represent the largest proportion of amputees) may also be under-studied in this area.

Figure 3e demonstrates the imbalance of socket type in the studies included in this review. The first (and only) study to utilise TSB sockets over more traditional PTB-total contact sockets in a study of pressure and alignment was Kang in 2006. Only one study has examined a hydrostatic socket (Courtney 2016, in comparison with a PTB design in one subject). Socket design has a clear impact on pressure distribution as the aims of PTB sockets and more recent designs are divergent, in terms of load position and concentration. This forms a significant limitation on the usefulness of results when they are substantially restricted to older socket/suspension technology. In total eight papers relied on old foot designs (i.e., a SACH foot), and seven on old socket designs.

Fatigue and learning effects were not accounted for in any included study. Tiredness is known to affect gait patterns in amputee walking [49, 50], and the studies presented describe extensive testing procedures with multiple recorded trials and numerous perturbations of socket conditions. Although the impact is mitigated somewhat by randomising the order of induced perturbations between participants, there will still be a residual impact in the ability to acclimatise to new conditions and in the comparison to the original ‘neutral’ alignment. The work by Sanders et al. in the evaluation of socket pressures across measurement sessions demonstrates that inter-session differences can be similar in magnitude to those induced by alignment changes: therefore greater attempts to assess the impact of fatigue within sessions would be a valuable addition to the pressure measurement literature.

Further to this, adaption to each intervention was also likely inadequate in all studies. Although the literature does not provide a firm recommendation for suitable acclimatisation time to alignment changes, a review of socket design changes found that allowed accustomisation times were around three months [49], although alignment changes are considered less significant than a socket design change. In Neumann’s 2009 review, acclimatization of less than 5 min was described as unsuitable. Most studies did not explicitly describe the adaptation time: the two that did (Sanders et al. [40] and Jia et al. [45]), restricted adaptation time to 5 min or less. Clearly this is a problematic issue that has to be balanced against the burden of participation, the fatigue of extended measurement sessions and the total time required for studies. Of the two studies to describe acclimatisation time, one (Sanders et al. [40]) deliberately minimised adjustment time, reportedly to maximise the changes in the measurement session.

Presumably due to the low participant numbers and short intervention sessions, attrition of participants during studies was low. The exception was Neumann, who recruited four participants but reported results for two: this was due to equipment failure in these cases. Only two studies (Sanders et al. [40], Sanders and Daly [42]) involved measurements in multiple sessions, in the former, two participants each in two sessions, and in the latter three participants, one each of whom took part in two, three and four sessions.

The reliability to outcomes was questionable in each included study. In particular, the methods for defining a suitable initial, ‘neutral’ or ‘optimal’ alignment that was then modified in the study was typically poorly described. Pearson et al. [37] reported initial alignment in terms of relative positions of components, and Kang et al. [44] in terms of the initial flexion angle of the socket relative to the pylon. The remaining studies merely reported that the alignment was considered suitable by the investigators—and so not sufficient to repeat the investigation.

It is recognised that the measurement or recording of initial/optimal alignment state is rarely completed in either research or clinical practice, and that there is a paucity of commercially-available equipment that is capable of such measurement. Nevertheless, the identification of a suitable alignment and the initial state of the prosthesis prior to intervention is an essential aspect in the repeatability and understanding of this form of study, and the lack of such information represents a threat to validity of the conclusions drawn. Numerous techniques and devices for the measurement of this have been published [51–54].

This threat was mirrored in the lack of quantification of the suitability of each alignment intervention by either the investigators or participants. The exception was the study by Neumann et al. [46], which used a standard question to rate the alignment acceptability at each stage (although the precise wording of the question was not included). Sanders et al. [40] completed fewer than intended interventions due to safety considerations of the produced alignments.

Included studies reflected the development in measurement instrumentation over time, from large diaphragm sensors to piston-based strain gauge sensors to force sensitive resistor arrays (Table 5). Although a detailed evaluation of sensor design is beyond the scope of this review (a review of this was recently published by Al-Fakih et al. [27]), methods improved in terms of convenience of application, sensing element resolution and in socket coverage. It is less clear that this always represented an improvement in sensor fidelity or reliability.

The introduction of sensor arrays has created a new issue in study reliability. As the arrays cover regions greater than the regions of interest on the interface, smaller ‘windows’ or subsections are identified and reported: the precise size and position of these windows is to some extent a subjective process. Several recent studies (Kang and Courtney) use such arrays, but do not report their methods for isolating subsections of the array for further analysis. Good practice was achieved by Neumann, who presented the precise size and location of the subregions of sensels included in their analysis, and differences between participants. However, suitable guidance for the meaningful selection of subregions of pressure measurement arrays does not appear to be available. Given that small changes in positioning of these windows can have substantial changes in the reported pressures (as seen in the similar application of foot plantar pressure measurement [55]) then rigorous justification for these analysis choices is essential for confidence in results.

The sampling rate of pressure measurement transducers varied between 50 and 200 Hz (and was unreported in three cases [37, 44, 47]: see Table 5). The literature is unclear as to a suitable collection frequency for socket interface pressures. Sanders et al. [39] identified high frequency components in their recordings, and ascribed them to factors such as rapid alterations in the configuration of the artificial foot, adjustment created from the contralateral side, muscle activation changing stump geometry and slip at the socket interface (and in probability some combination of these). Clearly, measurement of these features requires a sufficient sampling rate to acquire them: however detailed numerical evaluation of these was not presented. Sanders et al. used these conclusions as justification for their choice of a 175 Hz sampling rate in their 1998 paper [42]: this was the only other commentary on collection frequency in the selected articles. A specific recommendation on sampling rate is not possible on the evidence included within this study.

Five studies did not include sufficient detail on the calibration methods employed and so are marked as containing threats to validity [37, 39, 42, 44, 45]. In socket pressure measurement is a challenging process, and sensors are known to suffer from numerous limitations to performance [56], including but not limited to hysteresis, full scale error, temperature sensitivity, and, in the case of combined shear sensors, evidence of cross talk error between axes. For this reason it is important that authors report on the method and results of calibration of the sensors in use, or clearly reference work which does so.

Insufficient reporting of descriptive statistics was present in some studies. At a minimum, results should describe the average and some measure of variance of each reported numerical result, as doing so facilitates the future incorporation of results into meta-analyses.

Several publications did not report results with the required completeness. Some collected data but then failed to include it (e.g., Pearson, who reported collecting data on coronal plane changes in all participants but only presented results for a single subject [37]). For some, thorough presentation of data was only completed for some aspects of the intervention (e.g., Winarski and Pearson [38], which did not report gastrocnemius data, and more recently Neumann et al. [46], who collected pressure data in different alignment states but presented experimental data from neutral alignment only).

There is a commonplace issue with regards to limited space to include complete results, particularly with array-based systems due to the volume of data collected. Nevertheless, online appendices can accommodate extensive datasets, and authors should be encouraged to utilise these whenever possible.

Statistical significance was only evaluated in a few studies [40, 43, 44] (and absent entirely in the remainder). All studies that calculated significance used the traditional benchmark of a p value <0.05 to determine statistical significance, but provided no rationalisation beyond this. Also of concern was the universal lack of justification for the use of parametric statistical tests (i.e., Student’s t-tests). As the fundamental assumption in the use of these tests is that the results are consistent with a normal distribution, it is important for this choice to be numerically confirmed, using a Kolmogorov–Smirnov test for example [57]. The failure to report the results of such tests may mean that non-parametric equivalent tests would have been more appropriate in these analyses. As these tests are typically more stringent in confirming statistical significance, the confidence that can be held in the conclusions of the included studies is reduced.

Similarly, some statistical tests employed were also misused within included studies. Sanders [40] and Kang [44] used t-tests to evaluate statistical significance on large numbers of comparisons. A more appropriate tool (assuming normal distributions have been confirmed) when using multiple comparisons in an ANOVA test [58]. A related issue is the use of some correction factor for the boundary of when results meet statistical significance when multiple comparisons are made (e.g., a Bonferroni correction). Doing so reduces the chances of a type I error.

There was a slight trend for studies to improve in quality over time. In the past studies were typically held to lower editorial standards, and were in many ways restricted in terms of available instrumentation, knowledge of what is now standard practice in prosthetic research and the benefit of accumulated understanding. These studies have been graded for applicability of conclusions using the same standards as for contemporary research. The decision to treat pioneering research in the same way as modern work is because the applicability of results to current researchers should not depend on the era in which the results were obtained.

### Discussion of Evidence Statements

Extensive conclusions on the impact of alignment changes on prosthetic socket pressure are difficult to draw due to the significant inhomogeneity of measurement techniques and interventions reported. Nevertheless, there appears to be moderate evidence for a systematic and repeatable change in pressures on the anterior and posterior surface in response to sagittal rotational alignment alterations within individuals. Lower quality evidence supports the idea that although changing alignment does cause meaningful shifts in pressure patterns across the socket, these changes are particular to individuals and to socket designs. Evidence statements are presented in Table 9.

**Table 9:** Evidence summary with associated confidence

Confidence	Lead authors	Key conclusions
High	N/A	None
Moderate	Sanders (1998)	• The majority of measured sites demonstrate significant pressure changes with alignment modification, with an emphasis on the posterior surface • Compensations to one alignment change are not necessarily symmetrical in response to opposite alignment alterations
	Sanders (1999)	• Misalignment effects are similar in magnitude to within and between session variances in experienced participants
	Seelen	• Plantarflexion increases subpatellar pressure and decreases tibial end pressure. Dorsiflexion decreases subpatellar pressure and increases tibial end pressure
	Kang	• A/P realignment alters pressure distribution in a systematic and consistent manner, including significant changes at the subpatella and tibial end regions
Low	Pearson	• Greater sensitivity to angular changes than translation changes
	Sanders (1993)	• Wave form shape changes were not consistent across sites or across subjects
	Jia	• Duration of sub-maximal pressure alters significantly, as does the time-pressure integral (to a greater extent than peak pressure alone)
	Neumann	• Fitted linear regression models are potentially unique for individuals and also for socket designs and alignments
	Courtney	• Individual responses are evident to alignment changes and associated socket design

Key conclusions from included articles, grouped by confidence in conclusions. The papers by Winarski [38] and Zhang [41] did not draw conclusions suitable for inclusion

Moderate-rated evidence from Sanders (1999) indicates that the changes from alignment can be similar in magnitude to the variance assessed between measurement sessions. Socket pressure measurement is known to be subject to numerous confounding factors (e.g., stump volume change), and this may be one reason for the dearth of stronger evidence statements.

Several studies commented on the greater sensitivity to angular changes than pure translation. It seems likely that this is because rotational changes in the sagittal plane will also act to alter the effective limb length of the prosthesis. In turn this becomes more difficult for the amputee to effectively compensate for in their gait pattern (particularly given the short acclimatisation times reported in the included studies). No studies performed an additional correction for changes in prosthesis length.

Moderate evidence supports the biomechanical assumptions of early theoretical work in the field. In particular: increases in subpatellar pressure/decreases in distal posterior pressure in response to plantarflexion of the ankle and to socket extension and the opposite in response to ankle dorsiflexion/socket flexion. This is consistent with consideration of the socket as a pseudo-joint. The relative lack of consistency with regards the magnitude of these changes and the less reliable response to other changes is both a function of differences in transducer design (e.g., in the protrusion of sensing regions into tissue) and of the individual differences between residual limbs.

Although the majority of studies reported values of peak pressure change only, one study concluded that greater differences were evident in other measures of loading response, such as pressure time integral. It is possible that there is greater distinguishing power contained within the measurements of interface pressure than is suggested by the basic values reported by the majority of studies. Unfortunately, the limited nature of the reports of these data precludes detailed analysis.

In summary, although moderate evidence supports some gross changes in residuum pressure distribution in response to alignment modification, changes in these patterns seem to contain significant individual components. Therefore, although interpretation of pressure data between or across test subjects has restricted utility, pressure measurements collected with a particular participant may still have clinical use.

#### Study Limitations

This review faces some limitations. In particular, the papers were identified and evaluated in detail by the lead author only (although the discussion and conclusions were reached with the assistance of the rest of the authors). By restricting the language of accepted studies to English, some relevant work may have been disregarded by the analysis.

The reviewers remained unblinded to the authors and origins of the included research papers, with the potential effect that this could bias the consideration of the quality of each paper. Blinding was felt to be ineffective in this instance due to the low number of suitable studies, and that it would be substantially mitigated by the well-defined assessment criteria of the AAOP review process.

The AAOP technique also suffers from some limitations when applied to a review of this type. All the included studies were of the same design, which to some extent limits the differentiation of studies. It is also designed for primarily clinical studies, and is less well-suited to reviews where significant areas of interest include technical aspects of measurement. The technique also maintains some grading elements which are to an extent subjective—for instance EV8 where overstatement of conclusions may be scored differently. However, the authors feel that the method was able to distinguish studies by methodological quality effectively.

## Conclusions

A systematic search of the literature identified 11 studies that examined the changes in prosthetic socket pressure distribution with device alignment in unilateral transtibial amputees. Reports were highly inhomogeneous in methods of measurement, and significant gaps exist in measurement of many changes in alignment configurations. The majority of studies exhibited numerous shortcomings in design and description: the quality of evidence supporting the conclusions of included studies was never rated higher than moderate, and for most studies was considered low. In particular, the quality of socket fit, quantification of initial alignment and the suitability of modifications to alignment were poorly carried out. External validity was also poor—this was a function of study design (all included studies were classed as before and after studies with participants acting as their own control) and of typically poor statistical quality.

Some evidence statements with moderate confidence could be made: in particular, there appears to be a reliable change in proximal anterior and distal posterior pressures in response to sagittal plane rotation. However changes in pressure distribution across the residuum can be regarded as having a strong individual component, making comparisons of patterns across participants challenging. This is thought to be due to the differences between residual limbs in terms of size and composition, in addition to the variances in socket design and manufacture.

Future publications in this field should endeavour to better meet the AAOP guidelines for the presentation and design of prosthetics research, and to present sufficient detail in their results to enable future compilation into a meta-analysis. A greater awareness of the limitations of the measurement equipment under use is also essential, particularly as systems move from purely research tools into wider clinical practice. Given the paucity of extant research, the recent advances in the practicality of measurement techniques and the clinical importance of the topic, the authors recommend frequent updates of this literature assessment in order to support clinicians in understanding the consequences of their prosthesis design choices.

## Appendix: AAOP Assessment Criteria

### Internal Validity

- *IV1* comparison or control group used: not applicable,
- *IV2* groups formed by random assignment: not applicable,
- *IV3* groups comparable at baseline: not applicable,
- *IV4* groups handled in the same way: not applicable,
- *IV5* control/comparison group appropriate: not applicable,
- *IV6* intervention(s) not blinded,
  - Blinding not mentioned or not described,
  - Not blinded to participants only,
  - Not blinded to assessors only,
- *IV7* inclusion criteria not appropriate,
  - Inclusion criteria not described,
  - Pooled amputation aetiology too broad,
  - Pooled age range too broad (i.e., adults mixed with geriatric),
- *IV8* exclusion criteria not appropriate,
  - Exclusion criteria not mentioned or described,
  - Quality of socket fit not described,
  - Socket fit reported as loose or otherwise inappropriate,
  - Associated gait pathologies present,
- *IV9* protocol does not address fatigue and learning,
  - Fatigue and/or learning effects not mentioned or described,
  - Randomisation of intervention order not described,
  - Additional burden of measurement equipment not described,
- *IV10* protocol does not address accommodation and washout,
  - Adaption period to intervention not described,
  - Adaption period is mentioned, but likely to be inadequate (i.e., ≤5 min),
- *IV11* reporting of attrition,
  - Reasons for attrition not described,
  - Attrition greater than 20% of initial recruitment,
- *IV12* Attrition occurs between groups: not applicable,
- *IV13* outcome measures lack reliability,
  - Optimal/Initial alignment cannot be replicated,
  - Alignment interventions cannot be replicated,
  - Assessor judgement of alignment acceptability cannot be replicated,
  - Participant judgement of alignment acceptability cannot be replicated,
  - Instrument calibration is not described,
  - Quality of instrumentation is not described or referenced,
  - Load positions/sensor array windows cannot be replicated,
  - Description of instrument collection process is not adequate for replication,
- *IV14* statistical design and analysis is not appropriate,
  - Sample size/number of trials/number of observations is insufficient to calculate descriptive statistics,
  - Sample size/number of trials/number of observations is sufficient to calculate descriptive statistics, but these are not reported,
- *IV15* effect size is not reported,
  - Calculation of effect size is unreported,
- *IV16* tests of significance,
  - Tests of significance could be undertaken, but they are not reported,
  - Inappropriate statistical tests are reported,
- *IV17* statistical power,
  - Statistical power is not reported,
- *IV18* conflicts of interest,
  - Funding source or potential conflicts of interest are not reported,
- *IV19* editorial errors exist,
  - Contradictions are present in the report,
  - Elements crucial to the results are unreported or are unclear.

### External Validity

- *EV1* sample characteristics are not adequately described,
  - Individual variability amongst participants in the sample are not reported (minimum of age, gender, time since amputation and socket type),
- *EV2* sample population is not representative of the whole target (clinical) population,
  - Only experienced amputees are included,
  - Only older foot designs are included (e.g., SACH or single axis foot),
  - Only older socket designs are included (e.g., PTB with Pelite liner),
- *EV3* outcome measures are not adequately described,
  - Lack of descriptive statistics for inter-study comparisons,
  - Statistical significance of results are not reported,
  - Relevant data was collected but not presented in the report,
- *EV4* outcome measures validity issues,
  - Lack of blinding or randomisation of interventions,
  - Fatigue or learning effects remain uncontrolled,
  - Other sources of error exist (e.g., known poor socket fit),
- *EV5* intervention not adequately described,
  - Lack of quantification of initial/optimal alignment,
  - Lack of quantification of alignment perturbation,
- *EV6* threats to clinical significance or relevance from reporting,
  - Lack of discussion,
  - Lack of recommendations regarding suitable alignment,
  - High cost of instrumentation,
  - Complex mathematical or statistical models are required,
  - Reported results appear highly participant-specific,
  - Major unexplained within-individual outcome variation,
- *EV7* conclusions in context of existing literature,
  - Comparisons to previous literature are not discussed,
  - Conclusions appear to contradict other studies,
- *EV8* conclusions with respect to findings,
  - Appear to be biased (e.g., report positive but not negative findings),
  - Appear to overstate or exaggerate findings,
  - Contradict data in tables/figures/discussions elsewhere in the text.
